# Physicochemical characteristics of meat from capons derived from the crossing of conserved breed hens and meat roosters

**DOI:** 10.1016/j.psj.2023.102500

**Published:** 2023-01-13

**Authors:** J. Calik, J. Obrzut

**Affiliations:** National Research Institute of Animal Production, Department of Poultry Breeding, 32-083 Balice k. Krakowa, Poland

**Keywords:** capon, meat quality, sensorial evaluation

## Abstract

The study aimed to determine the effect of castration on physicochemical properties of meat from capons derived from the crossing of Rhode Island Red (R-11) hens and meat roosters. Subjects were 100 crossbred cockerels, which were randomly assigned to 2 groups of 50 each. Group I (control) consisted of intact cockerels and birds from group II were subjected to castration. The castration was performed at 7 wk of age under local anesthesia by a veterinarian. All birds were fed *ad libitum* with the same feed mixtures and placed on litter under optimal environmental conditions at a stocking density of 5 birds/m^2^. At the end of fattening (20 wk of age), 10 birds whose body weight was similar to group average, were selected from each group for slaughter. After slaughter, the birds were analyzed for dressing percentage and physicochemical parameters of breast and leg muscles, which were subjected to sensory evaluation. The results demonstrated that compared to intact cockerels, capons were characterized by significantly higher body weight, dressing percentage and carcass muscle content. Both the breast and leg muscles of the capons showed better physicochemical parameters and higher sensory score. It is therefore concluded that the birds derived from the crossing of Rhode Island Red (R-11) hens and meat roosters are good material for capon production, their meat is a product of distinctly higher quality.

## INTRODUCTION

In recent years, the growing demand for high quality poultry products has provided an opportunity to increase the importance of keeping native or locally adapted breeds of hens (Amorim et al., 2016; Obrzut et al., 2018; Calik et al., 2020). In Poland, 8 breeds (including 11 lines of laying hens) are under conservation, most of which are listed in the World Watch List (2000). Practical use of these populations in the extensive farming system is one of the methods for preserving biodiversity of these hens. According to Puchała et al. (2014), Krawczyk and Calik (2018), and Kwiecień et al. (2018), the conserved breeds as well as their crosses show phenotypic and production differences while providing products with good taste and dietary qualities. Sokołowicz et al. (2016) highlight that the quality of meat from native chicken breeds is significantly different from the meat of broiler chickens, mainly in terms of carcass and muscle color, fat content, muscle fiber thickness, tenderness, chemical, and sensory characteristics of meat, which are dependent on bird genotype, farming method, diet, and slaughter age. The intensification of laying hen production has caused the problem of surplus cockerels, which may be resolved by subjecting the cockerels to castration and fattening, especially since we currently observe a revival of Old Polish cuisine, in which capon meat was used to make exquisite dishes (Calik, 2014).

According to the Commission Regulation (EC) No 543/2008 of 16 June 2008, capons are “male fowl castrated surgically before reaching sexual maturity and slaughtered at a minimum age of 140 d.” Castration of cockerels is a very old practice, known long before the Christian era in China, Rome and Greece (Symeon et al., 2010,^2012^). Today this procedure is commonly performed in many countries of Asia, Europe and America, where native breeds of hens are mostly used for their production and capons are marketed as high quality products (Sirri et al., 2009; Amorim et al., 2016). The studies of Mahmud et al. (2013), Franco et al. (2016), and Calik et al. (2017,^2018^,^2020^) show that sterilized cockerels are characterized by higher body weight gains and better feed conversion compared to intact cockerels. This is accompanied by increased deposition of adipose tissue in the abdominal cavity and of subcutaneous (especially intramuscular) fat, which makes capon meat more juicy and tender, thus improving the taste properties (Chen et al., 2006; Miguel et al., 2008; Amorim et al., 2016; Calik et al., 2017; U-Chupaj et al., 2017). In our researches, rearing and fattening results of capons obtained from pure hen lines showed that they require longer rearing due to the slow rate of weight gain (Calik, 2015; Calik et al., 2015,^2017^). Therefore, we attempted to use capons derived from the crossing of Rhode Island Red (R-11) hens and meat roosters for production.

The objective of the study was to determine the effect of caponization on the physicochemical properties of meat from capons obtained by the crossing of Rhode Island Red (R-11) hens and meat roosters.

## MATERIALS AND METHODS

All the experimental procedures conformed to the requirements of the Local Ethics Committee for Animal Experimentation (no. 1121/2014) in Kraków, Poland. Subjects were crosses obtained by the crossing of Rhode Island Red (R-11) hens and meat roosters Ross 308. After weighing and individual tagging, one-day-old chicks were randomly assigned to two groups of 50 each. Control group I (50 birds in 5 pens with 10 birds per pen) consisted of intact cockerels and birds from experimental group II (50 birds in 5 pens with 10 birds per pen) were castrated. The castration was performed under local anesthesia by a veterinarian when birds were 7 wk of age and weighed around 600 g.

Birds were kept under optimal environmental conditions in a welfare friendly farming system:

• temperature: 32°C during the first days of rearing, gradually decreasing as birds aged to reach 17-20°C after 35 d of age,
• relative humidity: 65 to 70%,
• lighting regime: 20 to 40 lx up to 14 d, followed by 5 to 10 lx,
• housing system: litter, stocking density of 5 birds/m^2^, enriched environment (perches, additional bales of straw, scratching areas, sand baths, claw-trimming areas, grit).

During the rearing and fattening period (up to 20 wk of age), cockerels and capons were fed ad libitum with complete mixtures formulated at the National Research Institute of Animal Production: starter (1–7 wk), grower (8–16 wk) and finisher with 4% milk powder (17–20 wk). Table 1 presents the ingredient composition and the results of chemical analysis of the feed mixtures according to AOAC procedures (2000).

**Table 1:** Composition and nutrient content of the diets used in the experiment (kg/100 kg).

Component	Phase I d 1 to wk 7	Phase II wk 8 to 16	Phase III wk 17 to 20
Ground maize	41.35	40.45	36.00
Ground wheat	25.00	22.00	29.40
Ground triticale	-	5.00	6.00
Ground barley	-	5.00	6.00
Soybean meal	30.00	24.00	15.50
Milk powder (32.7% protein, 3.0% fat)	-	-	4.00
Ground limestone	1.25	1.30	1.15
Dicalcium phosphate	1.60	1.45	1.15
NaCl	0.30	0.30	0.30
Vitamin-mineral premix DKA-F (finisher) (0.5%), kg	0.50	0.50	0.50
Crude protein (g)	204	184	165
Metabolizable energy (MJ) (kcal)	11.92 2850	12.05 2880	12.18 2910
Lys (g)	10.3	8.90	7.50
Met (g)	3.10	2.85	2.60
Ca (g)	8.95	8.60	7.90
P available (g)	4.10	3.80	3.50

The health of birds and group feed intake were monitored during the experiment. At the end of fattening (20 wk), 10 birds (control group–selected from 5 pens of 2 pieces and experimental group II–selected from 5 pens of 2 pieces) whose body weight was similar to group average, were selected from each group for slaughter. Before the slaughter, the selected birds received no feed for around 12 h but had constant access to water. After the slaughter, the group of castrated birds was checked for castration success (removal of the testes) and then subjected to standard postslaughter processing (scalding, defeathering, evisceration). Next, a Minolta CR 310 device (Konica Minolta Holdings, Inc., Japan) was used to determine the color of carcass with skin. Chilled carcasses (24 h at 4°C) were subjected to simplified slaughter analysis according to Ziołecki and Doruchowski (1989) to determine dressing percentage with giblets, dressing percentage without giblets, and the percentages of breast and leg muscles, giblets (gizzard, liver, heart), bones, and abdominal fat.

Samples of the pectoral and leg muscles were taken from each whole poultry carcass, and the physico (10 pieces from each group) characteristics of the meat were assessed:

• acidity–determined 15 min (pH_15_) and 24 h (pH_24_–ultimate pH) postmortem using a CyberScan 110 pH meter equipped with a glass electrode for meat analysis (Eutech Instruments Pte Ltd/Oakton Instruments with Hamilton glass electrode);
• color–determined 24 h postmortem with the L*a*b* system (CIE, 2007), using a Minolta CR 310 reflectance colorimeter (Konica Minolta Holdings, Inc., Japan; light source D65, observer 2°) and reflective spectrophotometer, where L* means lightness, a positive a* value means redness, and a positive b* value means yellowness–1 measurement from the dorsal part, 2 measurements from the thoracic part, 2 measurements from the thigh part of the legs, while the muscle color is the mean of 2 measurements of the breast muscle and 2 femoral muscle measurements taken on the inner surface immediately after bone separation;
• drip and cook loss determination–the meat juice leakage was determined after storing the samples of breast muscles and thigh muscles for 24 h at +4°C. In order to do this, 80 g meat samples were collected from the thigh and the pectoral muscle which were then placed in tightly sealed containers and stored in a refrigerator.
• thermal loss was determined as the loss in weight of breast and thigh muscles during cooking. Eighty g samples were placed in plastic bags and cooked at 100°C for 14 min (breast muscles) and 16 min (thigh muscles) to an internal temperature of 78°C. After cooking, the samples were chilled at room temperature for 30 min and then in a refrigerator at 4°C for 45 min.
• water-holding capacity (WHC)–of breast muscles and leg muscles were determined using the Grau'a and Hamm'a method (1953), based on the amount of juice mechanically pressed from the sample onto a filter paper (Whatman, 1 Qualitative, Cat No 1001 917, UK Limited), and the leakage area was estimated using a planimeter (Haff Digital Polar Planimeter No. 301, Germany).
• shear value determination–the measurement of muscle tenderness was performed using the TA.XT.plus texture analyzer (Stable Micro Systems Ltd, Godalming, Surrey, GU71YU, UK). In order to do this, samples 10 mm in diameter and 30 mm in height were cut out from the cooked breast and thigh muscle (78°C). The collected sample was cut with a Warner-Bratzler knife (2 mms^−1^) in three places, perpendicularly to the course of muscle fibres, while the final measurement result was provided as a mean value.

Chemical analyses were performed at the Central Laboratory of the Institute. Samples of breast and thigh were collected from 5 birds of each group (control group–selected from 5 pens of 1 pieces and experimental group II–selected from 5 pens of 1 pieces) in order to determine the chemical composition, that is, the contents of total dry matter, total protein (Kjeldahl method) and crude fat (Soxhlet method) and collagen using the AOAC method (2000). Cholesterol content was determined using gas chromatography (Shimadzu GC-2010 Plus). Fatty acid content was determined by gas chromatography (VARIAN 3400 CX) using helium as a carrier gas and column Rtx 2330 (105 m). Injector temperature was 200°C and detector temperature 240°C. The samples were prepared by the method of Folch el al. (1957) using BF3/methanol methylation.

The sensory evaluation test was carried out on without salt or spices to an internal temperature of 80°C. After cooking, the muscle samples were cut into 1 × 2 cm pieces, protected with aluminum foil, and marked with an identification number. Next, the samples were served for tasting to a sensory panel consisting of 10 people. The team of people included experienced and specially trained employees of the Institute. The following parameters were taken into account in the evaluation: aroma, juiciness, tenderness and taste. A 5-point scale from 1 to 5 points (with an accuracy of 0.5 points) was used, with a rating of 5 being the best, and 1 being the worst. The study also included sensory evaluation of breast muscles and thigh muscles, according to the methodological assumptions of Baryłko-Pikielna and Matuszewska (2009).

The obtained results were verified and the significance of differences was specified using Student's t-test (Statistica 13.0). The statistical analysis involved the determination of arithmetic means with their standard errors (s.e.) for the traits analyzed in the study.

## RESULTS

Mortality and health-related culling ranged from 0.00 (group I) to 2.50% (group II), with feed consumption per kilogram of weight gain being 4.88 and 4.48 kg, respectively (Table 2). The results for body weight and slaughter analysis of the cockerels and capons are presented in Table 3. Mean body weight at 20 wk of age was significantly (*P* ≤ 0.01) higher (by 314 g) in group II (caponized birds), in which carcass weight loss during storage was also significantly (*P* ≤ 0.05) lower, by 0.34 percentage points (pp). Castrated birds also exhibited significantly (*P* ≤ 0.05 and *P* ≤ 0.01) higher dressing percentage with giblets and without giblets (1.63 and 1.33 pp). Capons were also characterized by significantly (*P* ≤ 0.05 and *P* ≤ 0.01) higher content of breast and leg muscles (by 1.67 and 1.34 pp). Group II was characterized by a significantly (*P* ≤ 0.01) greater percentage of internal organs (by 0.28 pp), in particular liver percentage. Our study revealed no significant differences in leg bone percentage. However, the analyzed groups differed significantly (*P* ≤ 0.01) in the content of abdominal fat, which constituted 2.15 and 3.31% of carcass weight, respectively. There were also large (*P* ≤ 0.01) differences in the color of carcasses, which in the caponized birds were lighter (L* = 72.39), more yellow (b* = 9.57), and less red (a* = 4.13).

**Table 2:** Mortality and health cullings and use feed on 1 kgs of body weight.

	Mortality and health cullings					
	Number of birds				%	Feed conversion ratio
Item	0–8 wk	9–12 wk	13–20 wk	Total	x ± s.e.	(kg/kg) x ± s.e.
Cockerels	-	-	-	0	0.00 ± 0.00	4.88 ± 0.05
Capons	1	-	-	1	2.50 ± 0.25	4.48 ± 0.06
P-value	-	-	-	-	0.359	≤0.01

Notes: s.e, standard error; x, mean value.

**Table 3:** Body weight and results of slaughter analysis.

Item	Cockerels x ± s.e.	Capons x ± s.e.	*P*-value
Live body weight (g)	4372 ± 45.80	4686 ± 58.85	≤0.01
Carcass weight loss during chilling (%)	1.47 ± 0.10	1.13 ± 0.08	0.034
Dressing percentage with giblets (%)	79.70 ± 0.31	81.33 ± 0.23	≤0.01
Dressing percentage without giblets (%)	76.66 ± 0.33	77.99 ± 0.22	0.011
Content in carcass:			
Breast muscles (%)	21.42 ± 0.31	23.09 ± 0.49	0.021
Leg muscles (%)	20.54 ± 0.12	21.88 ± 0.20	≤0.01
Offals (%)	3.04 ± 0.04	3.33 ± 0.06	≤0.01
Liver (%)	1.33 ± 0.04	1.56 ± 0.04	≤0.01
Gizzard (%)	1.27 ± 0.04	1.35 ± 0.05	0.213
Heart (%)	0.45 ± 0.02	0.42 ± 0.04	0.592
Leg bones (%)	5.08 ± 0.07	5.02 ± 0.06	0.581
Abdominal fat (%)	2.15 ± 0.16	3.31 ± 0.08	≤0.01
Carcass color:			
– L*	70.77 ± 0.23	72.39 ± 0.20	≤0.01
– a*	6.20 ± 0.39	4.13 ± 0.28	≤0.01
– b*	8.07 ± 0.21	9.57 ± 0.33	≤0.01

Notes: s.e, standard error; x, mean value.

Table 4 shows technological parameters of breast and leg muscles. The groups under study did not differ in muscle acidity measurements taken 15 min and 24 h after slaughter, and the decrease in pH between the measurements ranged from 0.40 to 0.46 points for breast muscles and from 0.52 to 0.54 points for leg muscles. In capons, both the breast and leg muscles were lighter (L*), more yellow (b*), and less red (a*). The breast and leg muscles of the capons also showed a significantly (*P* ≤ 0.01) lower drip loss after 24 h of storage (by 0.27 and 0.17 pp) and lower losses during heat treatment (by 2.61 and 4.94 pp). Furthermore, caponization had a positive effect on the tenderness of breast and leg muscles (by 2.84 and 3.96 pp) and on better water holding capacity (by 2.65 and 2.93 pp), with significant (*P* ≤ 0.01) differences between the analyzed groups (*P* ≤ 0.01).

**Table 4:** Technological parameters of breast and leg muscles.

Item	Breast muscles x ± s.e.			Leg muscles x ± s.e.		
	Cockerels	Capons	*P*-value	Cockerels	Capons	*P*-value
pH_15_	6.33 ± 0.02	6.30 ± 0.03	0.124	6.63 ± 0.02	6.65 ± 0.04	0.512
pH_24_	5.87 ± 0.02	5.90 ± 0.02	0.245	6.11 ± 0.03	6.11 ± 0.03	0.624
Drip loss after 24 h (%)	0.64 ± 0.02	0.37 ± 0.02	≤0.01	0.46 ± 0.03	0.29 ± 0.01	≤0.01
Thermal loss (%)	26.44 ± 0.49	23.83 ± 1.17	0.073	33.44 ± 0.65	28.50 ± 0.57	≤0.01
Color:						
- L*	60.10 ± 0.41	61.78 ± 0.52	0.035	46.40 ± 0.66	50.29 ± 0.95	0.010
- a*	10.68 ± 0.68	8.85 ± 0.31	0.039	18.33 ± 0.46	16.47 ± 0.61	0.041
- b*	7.56 ± 0.36	8.86 ± 0.33	0.028	7.01 ± 0.26	8.48 ± 0.36	0.011
Tenderness (N)	18.71 ± 0.18	15.87 ± 0.60	≤0.01	23.90 ± 0.59	19.94 ± 0.50	≤0.01
Water holding capacity (%)	18.02 ± 0.25	15.37 ± 0.39	≤0.01	18.79 ± 0.19	15.86 ± 0.39	≤0.01

Notes: s.e, standard error; x, mean value.

In the sensory analysis (Table 5), both the breast and leg muscles of the capons received higher scores in all the categories (color, aroma, juiciness, and tenderness) compared to the meat of intact cockerels, with significant differences between the analyzed groups (*P* ≤ 0.05 and *P* ≤ 0.01).

**Table 5:** Results of sensory analysis of breast and leg muscles.

	Breast muscles x±s.e.			Leg muscles x±s.e.		
Item	Cockerels	Capons	*P*-value	Cockerels	Capons	*P*-value
Aroma (pts.)	4.25 ± 0.15	4.65 ± 0.12	0.025	4.20 ± 0.16	4.65 ± 0.12	≤0.01
Juiciness (pts.)	4.20 ± 0.12	4.60 ± 0.10	≤0.01	4.20 ± 0.17	4.80 ± 0.10	≤0.01
Tenderness (pts.)	4.10 ± 0.17	4.61 ± 0.12	≤0.01	4.30 ± 0.19	4.85 ± 0.07	≤0.01
Flavor (pts.)	4.30 ± 0.14	4.75 ± 0.10	≤0.01	4.35 ± 0.13	4.85 ± 0.07	≤0.01

Notes: s.e, standard error; x, mean value.

Table 6 brings together the differences in basic chemical components between the muscles of cockerels and capons. In both the breast and leg muscles, the castration had a significant (*P* ≤ 0.05 and *P* ≤ 0.01) effect on increasing dry matter (by 1.05 and 1.90 pp), crude protein (by 0.91 and 0.77 pp), and crude fat (by 0.43 and 1.90 pp). In addition, the breast and leg muscles of the castrated birds had a significantly (*P* ≤ 0.05 and *P* ≤ 0.01) lower content of collagen (by 0.27 pp on average), while the differences in ash and cholesterol in the analyzed muscles were small and not significant.

**Table 6:** Results of effect of caponization on chemical analysis of the breast and leg muscles.

	Breast muscles x±s.e.			Leg muscles x±s.e.		
Item	Cockerels	Capons	*P*-value	Cockerels	Capons	*P*-value
Dry matter (%)	26.27 ± 0.13	27.32 ± 0.25	≤0.01	25.36 ± 0.20	27.26 ± 0.28	≤0.01
Crude ash (%)	1.11 ± 0.01	1.10 ± 0.01	0.423	1.08 ± 0.01	1.09 ± 0.01	0.658
Crude protein (%)	24.03 ± 0.21	24.94 ± 0.12	≤0.01	20.05 ± 0.10	20.82 ± 0.29	0.039
Crude fat (%)	1.23 ± 0.05	1.66 ± 0.06	≤0.01	4.03 ± 0.19	5.93 ± 0.37	≤0.01
Collagen [g/100g]	0.81 ± 0.04	0.53 ± 0.02	≤0.01	1.64 ± 0.12	1.38 ± 0.06	0.074
Cholesterol (mg/100 g)	0.57 ± 0.02	0.58 ± 0.01	0.775	0.86 ± 0.01	0.85 ± 0.01	0.494

Notes: s.e, standard error; x, mean value.

The results for fatty acid content in the analyzed muscles are shown in Table 7. The breast muscles of capons had a significantly (*P* ≤ 0.05) lower content of stearic (C_18:0_), linoleic (C_18:2n-6_), and γ-linolenic acids (_Gamma 18:3n-6_) and a higher content of oleic acid (C_18:1_). In general, the capons showed a significantly (*P* ≤ 0.05) higher content of monounsaturated fatty acids (**MUFA**) and lower content of polyunsaturated fatty acids (**PUFA**), mainly n-6 PUFA. The n-6 to n-3 PUFA ratio in the breast muscles ranged from 13.83 in capons to 16.25 in cockerels (*P* ≤ 0.05). The leg muscles of the capons had a significantly (*P* ≤ 0.05 and *P* ≤ 0.01) lower content of stearic (C_18:0_), linoleic (C_18:2n-6_) and γ-linolenic acids (_Gamma 18:3n-6_), a lower content of arachidic (C_20:4n-6_) and docosahexaenoic acids (**DHA**) as well as a higher content of palmitic (C_16:0_), palmitoleic (C_16:1_), and oleic acids (C_18:1_). Group II showed a significantly (*P* ≤ 0.01) higher content of MUFA and a lower content of PUFA, in particular n-6 PUFA. The n-6/n-3 PUFA ratio varied between 14.74 in capons and 19.21 in cockerels (*P* ≤ 0.01).

**Table 7:** Results of the fatty acid profile of breast and leg muscles (g/100g).

	Breast muscles x ± s.e.			Leg muscles x ± s.e.		
Fatty acids	Cockerels	Capons	*P*-value	Cockerels	Capons	*P*-value
C _14:0_	0.52 ± 0.039	0.47 ± 0.020	0.342	0.59 ± 0.037	0.60 ± 0.017	0.705
C _16:0_	24.28 ± 0.670	25.26 ± 0.213	0.201	22.45 ± 0.697	25.33 ± 0.394	≤0.01
C _16:1_	3.94 ± 0.607	5,55 ± 0.542	0.082	4.99 ± 0.905	8.04 ± 0.600	0.023
C _18:0_	8.69 ± 0.504	6.92 ± 0.502	0.037	9.16 ± 0.878	5.44 ± 0.281	≤0.01
C _18:1_	27.22 ± 1.394	32.61 ± 1.698	0.040	29.50 ± 2.001	36.69 ± 1.008	0.012
C _18:2n-6_	20.26 ± 1.171	17.02 ± 0.417	0.031	23.02 ± 1.582	18.54 ± 0.608	0.030
Gamma _18:3n-6_	0.38 ± 0.021	0.32 ± 0.013	0.040	0.36 ± 0.019	0.27 ± 0.013	≤0.01
C _18:3n-3_	0.99 ± 0.105	0.93 ± 0.05	0.641	1.14 ± 0.080	1.23 ± 0.070	0.394
C_22_	0.28 ± 0.026	0.26 ± 0.033	0.583	0.14 ± 0.017	0.11 ± 0.003	0.079
C _20:4n-6_	12.32 ± 1.559	9.58 ± 1.451	0.234	8.07 ± 0.993	3.36 ± 0.106	0.002
C _22:6n-3_(DHA)	0.99 ± 0.134	0.93 ± 0.137	0.762	0.46 ± 0.063	0.24 ± 0.009	≤0.01
∑ SFA	33.83 ± 0.320	32.96 ± 0.441	0.148	32.40 ± 0.416	31.57 ± 0.646	0.312
∑ UFA	66.17 ± 0.320	67.04 ± 0.441	0.148	67.60 ± 0.416	68.43 ± 0.646	0.312
∑ MUFA	31.17 ± 1.809	38.17 ± 2.026	0.033	34.51 ± 2.749	44.74 ± 1.079	≤0.01
∑ PUFA	34.99 ± 1.755	28.87 ± 1.687	0.036	33.09 ± 2.431	23.69 ± 0.711	≤0.01
∑ PUFA_n-6_	32.97 ± 1.722	26.92 ± 1.563	0.031	31.45 ± 2.391	22.17 ± 0.650	≤0.01
∑ PUFA_n-3_	2.03 ± 0.057	1.95 ± 0.126	0.578	1.64 ± 0.060	1.51 ± 0.068	0.203
PUFA_n-6/n-3_	16,25 ± 0.734	13.83 ± 0.193	0.013	19.21 ± 1.135	14.74 ± 0.373	≤0.01

Notes: s.e., standard error; x, mean value.

## DISCUSSION

Late maturing birds with a good rate of weight gain are suited to extensive systems, and the crossing of different hen breeds improves weight gains and muscling in the birds (Yin et al., 2013), which was also applied in our study. The obtained crossbred capons achieved over 40% higher body weight compared to the pure Rhode Island Red (R-11) line, which allowed their fattening period to be shortened by 4 wk (Calik et al., 2017). Similar results were reported by Krawczyk et al. (2018) for poulards derived from the crossing of native hen breeds and meat roosters.

The health of the analyzed birds during the rearing and fattening periods was at a good level. Caponizing losses reported in the literature vary from 5 to 20% according to body weight and age of the birds subjected to castration (Rikimaru et al., 2009). As a result of testosterone deficiency, castrated birds show changes in behavior and appearance (Chen et al., 2007; Shao et al., 2009), which was observed in the present study as well. Symeon et al. (2010) and Volk et al. (2011) indicated that lower physical activity of castrated birds improves feed conversion, which is closely related to higher weight gains and deposition of intramuscular fat. In Poland, the final period of capon feeding was highlighted centuries ago by using a “white diet” based on milk or whey (Calik, 2014). Therefore, for our study we formulated starter and grower diets (loose feeds) as well as finisher diet (pellets) supplemented with 4% milk, which was fed to both groups of the birds during the last 4 wk of fattening. Also Rikimaru et al. (2009) reported lower feed consumption in sterilized birds. In studies with poulards and capons, respectively, Krawczyk et al. (2019) and Calik et al. (2020) showed that adding 4% milk powder to the diets during the final fattening phase had a positive effect on reducing feed intake per kg weight gain, on increasing body weight, and, most importantly, on improving the sensory qualities of the meat.

Our results point to the positive effect of castration on the final body weight of the birds. This corresponds with the findings of Diaz et al. (2010), Kwiecień et al. (2015), Franco et al. (2016), Zawadzka et al. (2017), Calik et al. (2017), Kwiecień et al. (2018), Calik et al. (2020), and Hossen et al. (2021). In contrast, Shao et al. (2009), Symeon et al. (2010), and Murawska et al. (2019) observed no advantage of capons over cockerels in terms of body weight. The authors indicated that the disparity in body weight could be associated with the genetic origin of the birds, their diets, management system, sterilization and slaughter date. Our experiment showed a positive effect of cockerel sterilization on dressing percentage with and without giblets as well as on carcass muscling, in particular on the higher content of breast and leg muscles, which is consistent with Duran (2004), Mahmund et al. (2013), Kwiecień et al. (2015), and Calik et al. (2017,^2018^,^2020^). As reported by Rahman et al. (2004), Chen et al. (2006), Kwiecień et al. (2018), and Murawska et al. (2019), castrated birds have higher weights of internal organs (especially liver and gizzard) compared to intact birds, which was observed in the present study as well. Chen et al. (2007) indicate that liver is the main site of fatty acid synthesis in birds, and increased liver weight may be due to increased lipogenesis. Similarly to the studies by Tor et al. (2005) and Calik et al. (2020), the analyzed groups of birds did not differ in bone content. The positive effect of castration on bone strength was also highlighted by Adamski et al. (2016a), whereas Tomaszewska et al. (2016) demonstrated that caponization of cockerels of the native Greenleg Partridge hens had no effect on bone weight and length, but reduced mineral density of the bones. The fact that capons in our study were more fatty than intact cockerels is consistent with Sinanoglou et al. (2011), Amorim et al. (2016), and Hossen et al. (2021). These authors concluded that capons have not only a higher content of abdominal fat, but also that of subcutaneous and intramuscular fat, while the castration of cockerels reduces testosterone concentration and increases the capacity for lipogenesis and accumulation of lipids in the body, which, in turn, may influence carcass color. In general, capon carcasses were lighter (L*), more yellow (b*), and less red (a*), which was observed in our earlier experiments as well (Calik et al., 2017,^2020^).

Sterilization of the birds had no effect on the pH of breast and leg muscles, which is consistent with the findings of Amorim et al. (2016) and Adamski et al. (2016b). Furthermore, the capon breast and leg muscles were characterized by more beneficial water holding capacity as well as positively lower drip loss and thermal loss, which agrees with the results of our earlier studies with pure lines of hens (Calik et al., 2017,^2018^) and their crosses (Calik et al., 2020). In addition, the analyzed breast and leg muscles of the capons were lighter and more yellow, whereas capon muscles more red, which is in agreement with the results of Sirri et al. (2009), Franco et al. (2016), and Calik et al. (2017). Symeon et al. (2012) reported that muscle color is determined mainly by the content of myoglobin and intramuscular fat and depends on breed, sex, age, and physical activity of the birds, and these factors have a direct influence on meat acidity, which, in turn, is strictly related to water holding capacity. The water holding capacity of meat is one of the major aspects from both the technological and economic standpoint (Połtowicz and Doktor, 2012). The authors stressed that this trait has a significant influence on meat juiciness and tenderness as well as on changes in the meat water content during transport, refrigerated storage and heat treatment. Our study indicates that caponization had a significant effect on muscle tenderness. In the instrumental analysis, breast and leg muscles of the capons were more tender than the muscles of intact cockerels, which was observed in the studies by Sirri et al. (2009), Lin et al. (2013) and Calik et al. (2017,^2018^,^2020^) as well. Meat tenderness is dependent on the content, composition and structure of intramuscular connective tissue, but also on the degree of postmortem degradation of myofibril and cytoskeletal muscle fiber proteins (Gesek et al., 2017,^2019^; Wojtysiak et al., 2019). The authors stress that changes in the rate of protein degradation postmortem influence not only the muscle fiber structure, but also the physicochemical parameters of the meat.

In the sensory analysis, both the breast muscles and especially the leg muscles of the capons received higher scores for all the analyzed parameters, that is, aroma, juiciness, tenderness and taste, which is consistent with the findings of Amorim et al. (2016), Franco et al. (2016), Gesek et al. (2017), and Calik et al. (2020). These authors highlight that higher accumulation of fat in the muscles contributes to improved sensory traits, and such meat shows better flavor, juiciness and tenderness. Augustyńska-Prejsnar and Sokołowicz (2014) and U-Chupaj et al. (2017) underlined that of great significance is the fat found in the muscles, which reduces the drying of muscle tissue during heat treatment and enhances juiciness. Furthermore, the authors stressed that tenderness is expressed through the subjective sense of meat hardness, elasticity or springiness. Puchała et al. (2014), Obrzut et al. (2018), and Krawczyk et al. (2019) concluded that the meat from native breed birds has more intense aroma and better taste, while the concentration of taste precursors increases with the age of the birds, reaching the maximum after the onset of sexual maturity. In addition, the authors observed that leg muscles with higher in vivo activity show stronger aroma than less active breast muscles, which was observed in the current experiment as well.

Castration had no effect on the ash content of the analyzed muscle parts, which is consistent with the study of Amorim et al. (2016); in contrast, Franco et al. (2016) showed that its content was higher in cockerels. Higher protein content was noted in both breast and leg muscles of the capons. Also the results of Kwiecień et al. (2016), Calik et al. (2015), Franco et al. (2016), and Calik et al. (2020) show significantly higher protein content in the muscles of sterilized birds. Collagen may form up to 30% of proteins in the avian body, and its high content in the muscle connective tissue has an important effect on meat tenderness by reducing its quality (Janicki and Buzała, 2013). In addition, the collagen network, which increases in vivo with the animal's age in high activity muscles, causes the meat to become hard. Sokołowicz et al. (2016) reported that lower collagen content is observed in the meat of late maturing and castrated animals, which was confirmed in our experiment. In the analyzed samples of capon breast and leg muscles the collagen content was significantly lower (by around 35 and 15%, respectively) than in intact cockerels. Furthermore, the breast muscles of cockerels and capons contained, respectively, around 50 and 60% less collagen compared to thigh muscles, which was observed by Amorim et al. (2016) and Calik et al. (2020) as well. Our study found no significant differences between the analyzed groups of the birds in cholesterol content, which was also reported by Lin and Hsu (2013), Kwiecień et al. (2015), and Calik et al. (2020). Different results were obtained by Sirii et al. (2009) who showed higher cholesterol content in both breast and leg muscles Both the breast and leg muscles of castrated birds were characterized by significantly higher content of dry matter and fat, which corresponds with the results of Lin and Hsu (2013), Obrzut et al. (2018), and Calik et al. (2020).

As indicated by Diaz et al. (2012), Miguel et al. (2008), and Tor et al. (2002,^2005^), the content of fatty acids in bird muscles is influenced by genotype, diet and age of birds, and the difference in the composition of fatty acids between breeds may be associated with the different fat content in the muscles. In our study, both the breast and leg muscles of the sterilized birds had a significantly higher content of MUFA, in particular a significantly higher content of oleic acid, which agrees with the findings of Kwiecień et al. (2018). What is more, the castration procedure had no effect on the content of saturated fatty acids (SFA), which was also observed by Tor et al. (2005) with Penedesenca Negra hens and by Amorim et al. (2016) with two native breeds of hens: Amarela Portuguesa and Pedres Portuguesa. Different results were obtained by Kwiecień et al. (2018) for Polbar (Pb) hens, which had higher content of SFA, in particular palmitic acid. Also Sirri et al. (2009) noted higher SFA content in the breast muscles of capons without effects on the content of MUFA and n-3 and n-6 PUFA. Our study showed a dietetically favorable lower n-6/n-3 PUFA ratio in castrated birds, which was also reported by Franco et al. (2016) and Calik et al. (2017,^2020^).

## CONCLUSION

It is concluded from the present study that:

• compared to intact cockerels, capons had significantly higher body weight, dressing percentage, and carcass muscling.
• both the breast and leg muscles of the capons were characterized by a better tenderness and a better water holding capacity and received higher sensory scores.
• birds obtained from the crossing of Rhode Island Red (R-11) hens and meat roosters are good material for capon production, their meat is a product of distinctly higher quality.
